# Studies on Intermolecular Interaction of *N*-Glycidyltrimethyl Ammonium Chloride Modified Chitosan/*N*,*N*-Dimethyl-*N*-dodecyl-*N*-(2,3-epoxy propyl) Ammonium Chloride and Curcumin Delivery

**DOI:** 10.3390/polym14101936

**Published:** 2022-05-10

**Authors:** Cangheng Zhang, Yan Li, Shu Xing, Xiaodeng Yang, Jinrong Zhao, Qiaoyan Dong

**Affiliations:** 1 Shandong Provincial Key Laboratory of Fine Chemicals, Qilu University of Technology, Shandong Academy of Sciences, Jinan 250353, China; canghengzhang@163.com (C.Z.); shuxingcareer@sina.com (S.X.); 2 Rocket Force Sergeancy School, Weifang 260000, China; jrzhao72@sina.com; 3 Shandong Fangyan Biological Technology Co., Ltd., Jinan 250021, China; xiuwaihuizhong2011@163.com

**Keywords:** *N*-Glycidyltrimethyl ammonium chloride modified chitosan, *N*,*N*-Dimethyl-*N*-dodecyl-*N*-(1,2-epoxy propyl) ammonium chloride, intermolecular interaction, curcumin encapsulation and release

## Abstract

Chitosan has potential applications in many fields, due to its biocompatibility, biodegradability and reproducibility. However, the insolubility in water restricts its wide application. In order to expand the application of chitosan in the delivery of oil-soluble drugs and improve the efficacy of oil-soluble drugs, *N*-Glycidyltrimethyl ammonium chloride-modified chitosan (GTA-m-CS) and *N*,*N*-Dimethyl-*N*-dodecyl-*N*-(1,2-epoxy propyl) ammonium chloride (DDEAC), a kind of reactive surfactant, were synthesized and characterized by FTIR, NMR and XRD methods. The interactions between GTA-m-CS and DDEAC was studied by surface tension, viscosity, conductivity and fluorescence methods. The parameters, including equilibrium surface tension, critical micelle concentrations of DDEAC with different GTA-m-CS concentration, critical aggregation concentration of DDEAC, the amount of DDEAC adsorbed on GTA-m-CS, *p*c_20_ and *π*_cmc_ were obtained from the surface tension curves. The influence of temperature on the above parameters were evaluated. The degree of counterion binding to micelle and the thermodynamic parameters of the system were calculated from the conductivity curves. According to the change of conductivity with temperature, the thermodynamic parameters of micellar formation were calculated. The aggregation number of DDEAC molecules in GTA-m-CS/DDEAC aggregates were calculated from steady-state fluorescence data. Based on the experimental results, the interaction models between GTA-m-CS and DDEAC were proposed. The GTA-m-CS/DDEAC aggregates could be used as curcumin carries, and achieved sustained release.

## 1. Introduction

Chitosan is a deacetylated product of chitin and composed of 2-amino-2-deoxy-D-glucopyranose and residual 2-acetamido-2-deoxy-Dglucopyranose units, which has potential applications in food, daily chemicals, cosmetics, environmental remediation, drug delivery and many other fields, due to its excellent properties such as biocompatibility, reproducibility and biodegradability [1,2,3,4,5,6].

Chitosan/surfactant systems show complex behavior, and play an important role in cosmetic, food science and controlled drug delivery for their rich phase behavior at both the interface and in bulk [7]. The chitosan/surfactant complexes that formed under different interactions can be applied in different areas. Chitosan and double-chain anionic surfactant derived from lysine (77KS) could form negatively charged complexes, which could be used as a drug delivery system for amoxicillin on a model polydimethylsiloxane surface [2]. Chatterjee and Judeh [8] revealed that chitosan/anionic surfactants, including sodium dodecyl sluphate, sodium dodecylbenzenesulfonate, sodium cholate and sodium deoxycholate, could stabilize fish oil-in-water emulsions and control the fish oil sustained release. The chitosan/sodium dodecyl sulfate could also form chitosan-surfactant-core-shell at sodium dodecyl sulfate concentration much larger than its critical micelle concentration, which could adsorb malachite green as much as 360 mg/g due to the asdsolubilization of malachite green on surfactant bilayer [9]. The result is consistent with that obtained by Bharmoria et al. [10]. Bharmoria et al. revealed that the interaction between chitosan and surfactant varies with the concentration of surfactant. While surfactant concentration is lower than critical aggregation concentration, the chitosan/surfactant complexes were induced by ion dipole, electrostatic and hydrophobic interactions. At a surfactant concentration higher than the critical micelle concentration, electrostatic and hydrophobic interactions play a key role in the formation of chitosan/surfactant complexes [10]. The results are consistent with those obtained from the chitosan/sodium lauryl ether sulfate system [11]. Both the sodium lauryl ether sulfate and chitosan co-adsorbed on the air/water interface when the concentration of sodium lauryl ether sulfate was lower than critical aggregation concentration, and almost all the sodium lauryl ether sulfate occupied the interface when its concentration was larger than critical micelle concentration. The two adsorption processes corresponded to different dynamic behaviors as a function of sodium lauryl ether sulfate concentration [11]. The region of hydrophobic interactions is linearly dependent on the concentration of chitosan, and the maximum mass ratio of chitosan to sodium lauryl ether sulfate is 1/3 by the appearance of precipitate [12]. Senra et al. studied the intermolecular interaction with diffusion DOSY NMR technique, and detected that the surfactant molecules were not associated with chitosan as soon as the ionic charge ratio was larger than 1 [13]. Many of the systems were operated under acidic conditions due to the water insolubility of chitosan, which limits its wide application.

There are amenable functional groups such as primary amine (NH), primary and secondary hydroxyl group (OH) in chitosan monomer [5]. To expand the application of chitosan, chemical modification without disturbing its degree of polymerization has been performed. For example, Piegat and coauthors prepared *N*,*O*-acylated chitosan derivatives, and found that these hydrophobical chitosan derivatives could serve as oil-soluble active compounds for their stabilization of oil-in-water emulsification [14]. Elsaid et al. prepared *O*-octanoyl-chitosan-polyethylene glycol, a kind of amphiphilic chitosan derivate, which could form micelles with average particle size of 37.41 nm at a concentration of 16.6 μmol/L at 20 °C. The micelles with rapamycin entrapment efficiency of 85.6% and loading efficiency of 16.3% showed a high scleral retention and successful permeation [15]. *O*-Hydroxypropyl-*N*-alkyl chitosan, with critical micelle concentrations and hydrophile-lipophile balance values, range from 0.016 g/L to 0.05 g/L and 5.33 to 13.89, depending on the length of alkyl chains, and displayed good foam characteristics and emulsibility [16]. *N*-Succinyl-*O*-(2-hydroxyl) dodecyl ether chitosan could reduce the equilibrium surface tension of water to a minimum value of 32 mN m^−1^, and its critical micelle concentration is 5.72 mmol/L [17].

Burr et al. [18] synthesized cationic alkylated chitosan with glycidyltrimethylammonium chloride and analogue 3-chloro-2-hydroxypropyl-*N*,*N*,*N*-dimethyllaurylammonium chloride containing C12 alkyl chain successively, which could form viscoelastic gels in the presence of sodium dodecyl sulphate. These chitosan derivatives could be used in the range of cosmetics, pharmaceutical and personal care. Hydrophobic interaction between the hydrocarbon chains of hydrophobic chitosan and non-polar tails of surfactant dominant the formation of hydrophobic chitosan/surfactant complexes. Pérez-Gramatges et al. [19] reported that about 45,000 polyoxyethylene (8) nonylphenol ether molecules were bonded on one molecule of hydrophobic chitosan, and hydrophobic interaction between hydrophobic chitosan and polyoxyethylene (8) nonylphenol ether was the dominant force in the formation of complexes compared to hydrogen-bonding and dipolar interactions. The hydrophobic interaction also enhanced the adsorption of hydrophobic chitosan/surfactant complexes at the air/water interface. Choi and coauthors revealed that *N*-[(2-Hydroxyl)-propyl-3-trimethyl ammonium] chitosan chloride (HTCC) could stabilize and improve encapsulation efficiency of nanostructured lipid carriers for poor water-soluble indomethacin, and show good sustained release property [20]. In previous work, *N*-[(2-Hydroxyl)-propyl-3-trimethyl ammonium] chitosan chloride (HTCC) [21] has been synthesized by nucleophilic substitution method in ionic liquid, the reaction mechanism was studied by density functional theory calculations [22]. A first-order reaction and the reaction kinetics equation of $\ln\left(-\frac{dC_{-NH_{2}}}{dt}\right)=-0.21+1.1\ln C_{-NH_{2}}$, and the relationship between the rate constant of chemical reaction and temperature, lnk=−4.11/T+11.06 were revealed [23].

To expand the application of chitosan in oil-soluble drug delivery, in the current work, *N*-glycidyltrimethyl ammonium chloride modified chitosan (GTA-m-CS) and *N*,*N*-Dimethyl-*N*-dodecyl-*N*-(1,2-epoxy propyl) ammonium chloride (DDEAC), a kind of reactive surfactant, were synthesized and characterized. The interaction between GTA-m-CS and DDEAC was studied by surface tension, viscosity, conductivity and fluorescence methods. The parameters such as critical micellar concentration, surface tension, surface pressure and adsorption capacity of DDEAC on GTA-m-CS molecules, thermodynamic parameters and composition of micelles were calculated. Based on the results, the interaction models between GTA-m-CS and DDEAC were proposed. Finally, the GTA-m-CS/DDEAC aggregates were used to encapsulate curcumin, whose release behaviors were studied. The combination of the two compounds can endow the drug delivery system with better properties, especially antibacterial activity. The work will provide insight into the potential application of GTA-m-CS/DDEAC system in drug delivery.

## 2. Experimental

### 2.1. Reagents

Chitosan, with numerically average molecular weight of 5.2 × 10^5^, purity higher than 95% and degree of deacetylation of 91.2%, was purchased from Chengdu Xiya Reagent Co., Ltd., (Chengdu, China). Glycidyl trimethyl ammonium chloride (GTA) was purchased from Adamas Reagent Co., Ltd., (Shanghai, China). *N*,*N*-dimethyl-*N*-dodecylamine (A.R.) was purchased from Chengdu Xiya Reagent Co., Ltd., (Chengdu, China). 1-Amino-3-methylimidazolium chloride (AmimCl, with purity higher than 99%) was supplied by Lanzhou Institute of Chemical Physics, Chinese Academy of Sciences (Lanzhou, China). Anhydrous ethanol (A.R.) and hydrochloric acid (A.R.) were purchased from Tianjin Fuyu Fine Chemicals Co., Ltd., and acetone (A.R.) was purchased from the Fine Chemical Plant of Laiyang Economic and Technological Development Zone.

**Scheme 1:**
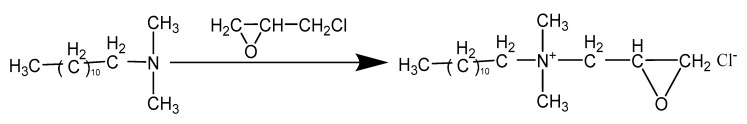

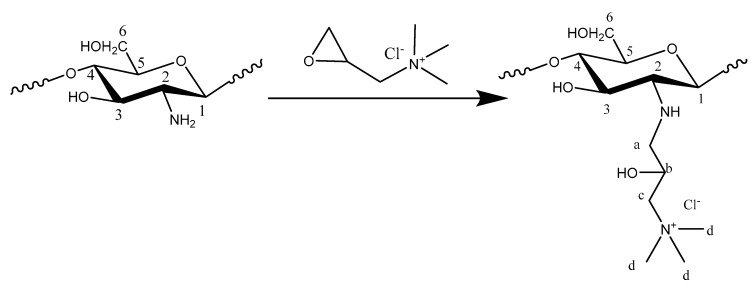
Schematic diagram of synthesizing DDEAC and GTA-m-CS molecular structure.

*N*-Glycidyltrimethyl ammonium chloride-modified chitosan (GTA-m-CS) was synthesized according to the method reported in previous works [21,24]. *N*,*N*-dimethyl-*N*-dodecyl-*N*-(2,3-epoxy propyl) ammonium chloride (DDEAC) was synthesized in our laboratory. The synthesizing method is the same as that reported in the previous work, using epichlorohydrin as both a solvent and reactant [25]. The reaction equations are shown in Scheme 1.

### 2.2. Characterization of GTA-m-CS and DDEAC

The chemical structure of GTA-m-CS was characterized on Shimadzu Prestige-21 FTIR spectrometer (Shimadzu, Japan) from 4000 to 400 cm^−1^ and Bruker Advance II 400 spectrometer (Bruker, Switzerland), respectively. The microstructure of GTA-m-CS was also characterized on an AXS D8-ADVANCE X-ray diffractometer (Bruker, Germany). The degree of substitution (DS) of GTA-m-CS was also calculated according to Equation (1)

(1) $$\mathrm{DS}=\frac{I_{{\mathrm{CH}}_{3}}/9}{I_{H_{1}}}\times 100$$

where *I*_*CH*_3__ and *I*_*H*_1__ were the integrals of *–CH*_3_ and *C*_1_-*H* in GTA-m-CS, respectively.

The molecular structure of DDEAC was characterized by ^1^H NMR, and the content of epoxy value was measured by perchlorate-tetraethyl ammonium bromide method.

### 2.3. Solution Froperties of GTA-m-CS/DDEAC

The aggregation behavior and intermolecular interactions between GTA-m-CS and DDEAC were studied by equilibrium surface tension, Ubbelohde viscosity, conductivity, steady state fluorescence and fluorescence quenching methods.

The equilibrium surface tensions were obtained on a K12 processor tensiometer (Krüss Co., Sable, Germany) using the ring method.

The viscosities were measured by an Ubbelohde viscosity with a capillary radius of 0.46 mm. The reduced viscosity was calculated.

The conductivities were performed on a low-frequency conductivity analyzer (DDS-307, Shanghai Precision and Scientific Instrument Co., Ltd., Shanghai).

The steady-state fluorescence was determined on a Hitachi F-4600 fluorescence spectrophotometer. A 1.0 cm quartz cell was used. Pyrene was used as the fluorescence probe, and its concentration was 1.0 × 10^−6^ mol/L in all determined systems. An excitation of 335 nm was used, and an emission spectra were collected from 355 to 500 nm. The fluorescence intensity ratio of the peak at 373 nm (signed as *I*_1_) to that of 383 nm (signed as *I*_3_) was calculated.

The micelle aggregation number (*N_agg_*) was calculated according to Equation (2) [26]

(2) $$\ln(\frac{I_{0}}{I})=\frac{N_{agg}C_{Q}}{C_{s}-cmc}$$

where *I*_0_ and *I* are pyrene fluorescence intensities at a wavelength of 373 nm with and without a quencher (benzophenone), and *CQ* and *Cs* are the molar concentrations of the quencher and surfactant, respectively.

The concentrations of GTA-m-CS were 400, 800, 1200, 1600 and 2000 mg/L, and those of DDEAC were ranged from 0.005 to 5 mmol/L. The temperature was fixed at 20 °C, unless there is specification.

### 2.4. Curcumin Encapsulation and In Vitro Releasing

The GTA-m-CS/DDEAC solution with concentrations of GTA-m-CS 1200 mg/L and DDEAC 3 mmol/L was used to load curcumin at room temperature. The method is similar to our previous work [27] under the assistance of ultrasonic vibration. The curcumin encapsulation efficiency (CEE) was calculated according to Equation (3)

(3) $$\mathrm{CEE}=\frac{w_{1}}{w_{2}}\times 100\%$$

where *w*_1_ and *w*_2_ are the weight of curcumin encapsulated in aggregates and initial weight of curcumin, respectively.

The hydrodynamic diameter and zeta potential of GTA-m-CS/DDEAC aggregates were measured on a Zetasizer Nano ZS90 particle size analyzer (Malvern, England) at 25 °C. Each sample was repeated three times.

The morphologies of GTA-m-CS/DDEAC aggregates with and without curcumin and DDEAC micelles with curcumin were observed on a transmission electron microscope (Tecnai-12) at 100 kV. The micelles were negatively stained with phosphotungstic acid and dried with an infrared lamp. The fluorescence confocal microscope (Axio Scope. A1, Germany Carl Zeiss Company, Germany) was used to observe the curcumin-loaded micelles after the curcumin-loaded GTA-m-CS/DDEAC solution was being dropped on a glass sheet.

The release behavior of curcumin from GTA-m-CS/DDEAC aqueous solution was determined at 25 °C. Typically, GTA-m-CS/DDEAC freeze-dried powder of 120 mg was weighed and dissolved in 100 g distilled water. Thus, the concentration of GTA-m-CS is 1200 mg/L, which is the same as that of curcumin being encapsulated. At different time intervals, 2.0 mL of release solution was taken out, while an equal volume of deionized distilled water was added to the mother solution. Equal volume of DMSO was added into the release solution of 2.0 mL, which UV absorption spectrum was measured.

Before the determination of the released curcumin concentration, the standard working curve was plotted.

## 3. Results and Dissolution

### 3.1. Molecular Structures of GTA-m-CS and DDEAC

**Figure 1:**
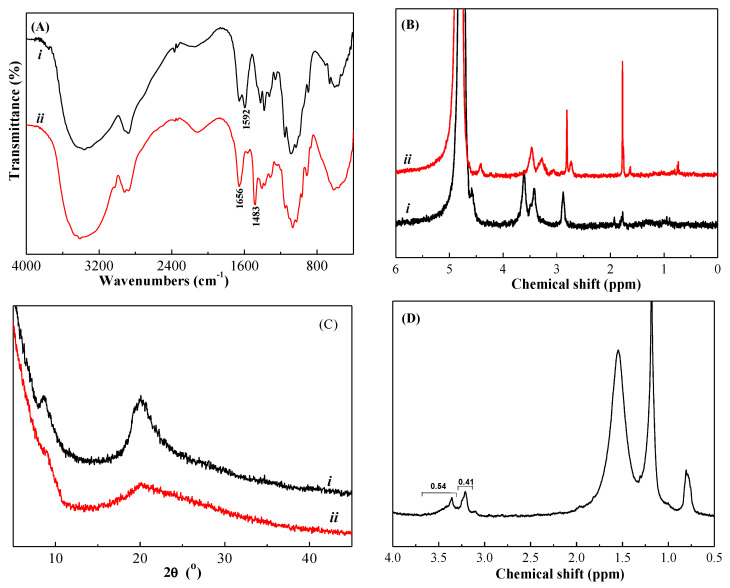
FTIR (**A**), ^1^H NMR (**B**) spectra and XRD patterns (**C**) of chitosan (*i*) and GTA-m-CS (*ii*) and ^1^H NMR spectra of DDEAC (**D**).

Figure 1A shows that the spectrum of GTA-m-CS is similar to that of chitosan. There are two strong peaks at 1656 cm^−1^ and 1483 cm^−1^ in spectrum *ii*, and a strong peak at 1592 cm^−1^ in spectrum *i*. The peak at 1656 cm^−1^ in spectrum *i* is very weak. According to previous result [24], the peaks at 1656 cm^−1^ and 1592 cm^−1^ belong to $v_{N-H}$ of tertiary amine and primary amine, respectively. The weakening of the peak intensity at 1592 cm^−1^ and the strengthening of peak intensity at 1656 cm^−1^ indicate that the nucleophilic substitution reaction occurred at the –NH_2_ group. The appearance of the peak at 1483 cm^−1^ is evidence of nucleophilic substitution, as it belongs to $v_{C-H}$ of $-{N(\mathrm{CH}}_{3}{)}_{3}^{+}$.

The ^1^H NMR spectra of chitosan and GTA-m-CS was shown in Figure 1B. The characteristic ^1^H NMR peaks of chitosan skeleton are observed at 4.6 (H_1_), 3.8–3.3 (H_3_, H_4_, H_5_, H_6_, H_6_′) and 2.9 ppm (H_2_). The chemical shift of GTA-m-CS are similar to those of chitosan, except the peaks at 2.8, 4.4, 3.2 and 3.02 ppm, which belong to methylene protons (H_a_), methane protons (H_b_), methylene protons (H_c_) and *N*,*N*,*N*-trimethyl protons (H_d_) [28], respectively. This confirms the formation of GTA-m-CS. The DS of GTA-m-CS calculated from Equation (1) is 36.6%.

The XRD pattern (Figure 1C) shows two diffraction peaks for chitosan: one at 2θ = 9°, belonging to the synergistic effect of (001) and (100) planes; and the other at 2θ = 20°, belonging to the synergistic effect of (101) and (002) planes. For GTA-m-CS, the diffraction peak at 2θ = 9° almost disappears, and the peak at 2θ = 20° becomes lower and broader. This means the decrease of crystalline degree after the introduction of GTA on chitosan [29].

For DDEAC (Figure 1D), the chemical shifts at 0.807, 1.193, 1.541, 3.212 and 3.358 ppm are assigned to –CH_3_, –CH_2_–, CH_3_CH_2_–, –N(CH_2_)– and –N(CH_3_), respectively. The area ratio of the peak at 3.212 to that of 3.358 ppm is 0.89, approximately equal to the ratio (0.67) of hydrogen atom numbers in –N(CH_2_)– and –N(CH_3_), demonstrating the synthesis of DDEAC. The epoxy value measured by the perchlorate-tetraethyl ammonium bromide method was 30.06%.

### 3.2. Surface Tension

**Figure 2:**
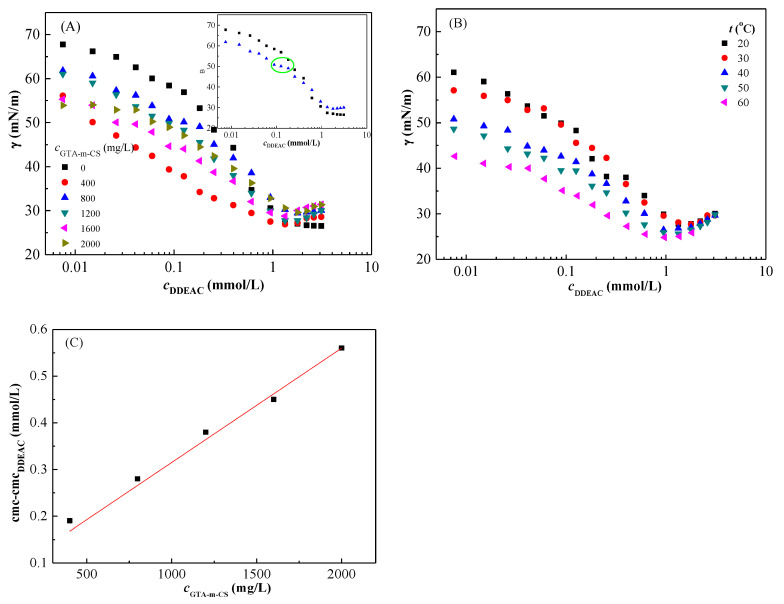
Plots of surface tension of GTA-m-CS/DDEAC system with different concentration of GTA-m-CS at 20 °C (**A**), with GTA-m-CS concentration of 1200 mg/L with different temperature (**B**) vs. DDEAC concentration, and linear relationship between cmc-cmc_DDEAC_ and *c*_GTA-m-CS_ (**C**).

DDEAC is a kind of cationic surfactant with reactivity, and its surface activity is higher than that of dodecyltrimethyl ammonium chloride [25]. Surface tension is one of the important methods in studying the interaction between macromolecules and surfactants. The relationship between GTA-m-CS solution and DDEAC with different concentration at 20 °C is shown in Figure 2A. The concentrations of GTA-m-CS are 0, 400, 800, 1200, 1600 and 2000 mg/L. The surface tension of GTA-m-CS decreases from 63 to 50 mN/m with concentration increasing from 400 to 2000 mg/L, meaning GTA-m-CS has certain surface activity. For GTA-m-CS/DDEAC systems, the surface tension decreases quickly with increasing DDEAC concentration until the surface tension keeps almost constant. The significantly decrease of the surface tension is caused by the adsorption of DDEAC on the surface. When the surface adsorption is saturated, the surface tension keeps constant and the DDEAC molecules begin to form micelles in the bulk phase. The concentration corresponding to the initial constant surface tension is called critical micelle concentration (cmc) [30]. The surface tension at cmc is recorded as γ_cmc_ in mN/m.

There are two platforms on GTA-m-CS/DDEAC curves, as shown in the insert of Figure 2A (green circle), which is different from that of DDEAC. The initial concentration of the first platform is critical aggregation concentration (cac), indicating the beginning of the interaction between GTA-m-CS and DDEAC and the formation of GTA-m-CS/DDEAC complexes [31,32,33]. At this platform, the DDEAC molecules adsorb on GTA-m-CS, keeping the surface tension almost constant. The value of cac is related to the properties of macromolecule and surfactant. The cac values of GTA-m-CS/DDEAC systems are all ca. 0.088 mmol/L. The formation of polymer/surfactant complexes has been confirmed by transmission electron microscopy and steady shear rheological methods [34].

After the saturated adsorption of DDEAC on GTA-m-CS molecules, the DDEAC molecules continue to adsorb on the solution surface, reducing surface tension until their adsorption on solution surface is saturated. The concentration corresponding to the saturated adsorption on surface is critical micelle concentration (cmc), and the corresponding surface tension is equilibrium surface tension (γ_cmc_). With a further increase of DDEAC concentration, the micelles are formed. The cmc and γ_cmc_ values of GTA-m-CS/DDEAC system are listed in Table 1, both of which increase with GTA-m-CS concentration. It is ascribed to the hydrophobic interaction between GTA-m-CS and DDEAC [33]. At a high concentration of GTA-m-CS, the GTA-m-CS molecules affect the adsorption and close arrangement of DDEAC molecules on the surface, inhibiting the further reduction of equilibrium surface tension (γ_cmc_).

The amount of DDEAC absorbed on GTA-m-CS could be calculated from the linear relationship of cmc_(GTA-m-CS)_-cmc_(DDEAC)_ vs. *c*_GTA-m-CS_ (Figure 2C), and the slope is the amount of absorbed DDEAC [24]. The fitted linear equation is as Equation (4), from which the absorbed amount at 20 °C is calculated as 2.45 × 10^−4^ mmol/mg.

(4) $$y=2.45\times 10^{-4}x+0.07(R^{2}=0.9975)$$

Two parameters of *p*c_20_ and *π*_cmc_ could be obtained from surface tension curves. The means of c_20_ is that the concentration of surfactant needed to decrease the surface tension by 20 mN/m, ${pc}_{20}=-{\mathrm{logc}}_{20}$. This concentration is the minimum one that surfactant reaches a saturation adsorption on the air/water interface. That is, *p*c_20_ reflects the efficiency of surfactant reducing surface tension. The values of *p*c_20_ are listed in Table 1, which show that GTA-m-CS/DDEAC systems are more effective in reducing surface tension. It ascribes to the interaction between GTA-m-CS and DDEAC, and the adsorption of GTA-m-CS on the air/water interface.

The parameter of *π*_cmc_ is the difference between the surface tension of solvent (γ_0_) and surfactant solution at cmc (γ_cmc_), reflecting the ability of reducing surface tension. The values of *π*_cmc_ in Table 1 show that the surface tension reducing ability of GTA-m-CS/DDEAC systems is almost equal to that of DDEAC system.

With the temperature increasing from 20 °C to 60 °C, the cmc values of GTA-m-CS (1200 mg/L)/DDEAC system decrease from 1.51 mmol/L to 0.83 mmol/L, and the surface tensions decrease from 27.78 mN/m to 24.63 mN/m as shown in Figure 2B and Table 2. This is attributed to the improved molecular thermal motion of both GTA-m-CS and DDEAC molecules [35]. Meanwhile, a high temperature promotes the adsorption of DDEAC molecules on the solution surface and decrease of surface tension [36]. The result is consistent with that of GMAC-m-CS/C14mimBr [24].

### 3.3. Viscosity

**Figure 3:**
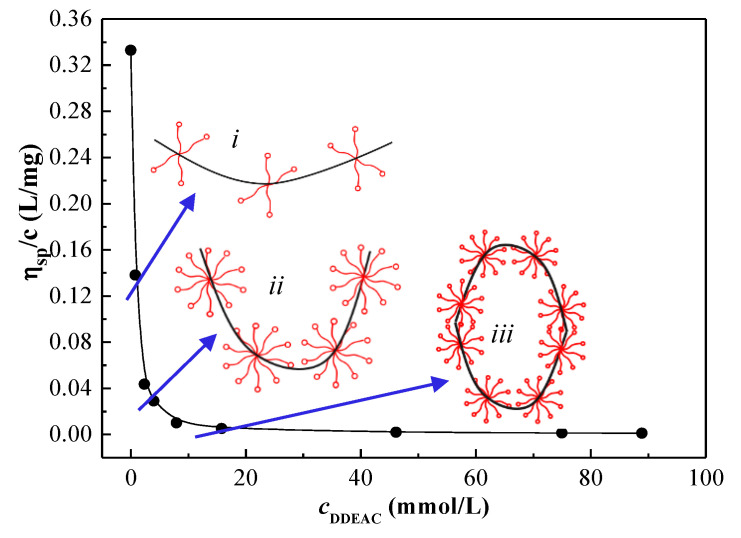
The relationship between reduced viscosity of GTA-m-CS/DDEAC and DDEAC concentration and schematic diagram of GTA-m-CS/DDEAC interaction (the insert). *i*—absorption of DDEAC molecules on GTA-m-CS molecule, *ii*—the formation of GTA-m-CS/DDEAC complexes, and iii-interaction between GTA-m-CS/DDEAC complexes.

To determine the intermolecular interaction between GTA-m-CS and DDEAC, the viscosity of GTA-m-CS (800 mg/L)/DDEAC system is measured at 20 °C, as shown in Figure 3. The reduced viscosity decreases quickly from 0.33 to 0.01 L/mg when the DDEAC concentration is increased from 0 to 8.0 mmol/L, and then with further increasing DDEAC concentration to 90 mmol/L, the reduced viscosity reduces slightly. The phenomenon is typical for polyelectrolyte solutions [37]. It is known that the reduced viscosity of pure polyelectrolyte solution decreases while its concentration is increased, because the macromolecular chains are expanded at a low concentration, and are overlapped at a high concentration [37]. For GTA-m-CS/DDEAC system, the decrease of reduced viscosity is ascribed to the adsorption of DDEAC on GTA-m-CS, and the decrease of intermolecular repulsion. The schematic diagram of GTA-m-CS/DDEAC interaction is shown in the insert of Figure 3. 

### 3.4. Conductivity

**Figure 4:**
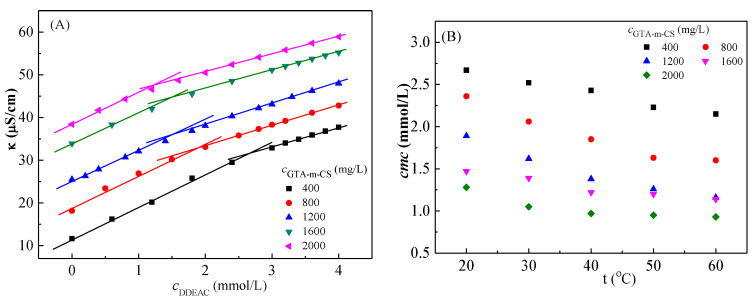
Plots of conductivity (**A**) and cmc (**B**) vs. concentration of DDEAC at different GTA-m-CS concentration and temperature.

Conductivity is one of the mostly used method in studying the aggregation behavior of surfactant [38]. The plots of conductivity vs. concentration of DDEAC in GTA-m-CS/DDEAC solutions are shown in Figure 4. The concentrations of GTA-m-CS are 400, 800, 1200, 1600 and 2000 mg/L, and the temperature ranges from 20 to 60 °C. All the conductivity curves are divided into two parts by one breakpoint. The breakpoint is the cmc. Before the cmc, the conductivity is from the contribution of the free ions of GTA-m-CS and DDEAC, and that of after the cmc is from the GTA-m-CS/DDEAC micelles. The charge transporting capability of the micelles decrease, and counter-ions bounded to the micelles reduce the conductivity [39]. The cmc values obtained from conductivity are listed in Table 3, which are different from those obtained from surface tension curves. It is due to the different testing principles and the fact that charged GTA-m-CS contributes to the conductivity of the system.

The variation of cmc values of GTA-m-CS/DDEAC systems with temperature at different GTA-m-CS concentration is shown in Figure 4B. At a constant temperature, the cmc values of GTA-m-CS/DDEAC decrease with increasing GTA-m-CS concentration. While the cmc values decrease with increasing temperature. Temperature shows two effects on the aggregation behavior of surfactant. On one hand, the hydration layer around surfactant molecules is destroyed while increasing temperature, which is beneficial to the aggregation of surfactants. On the other hand, the molecular movement is strengthened at high temperature, hindering the aggregation of surfactants [40]. The aggregation behavior of DDEAC is more influenced by the second factor. At a high temperature, the electrostatic repulsion between polyelectrolyte molecules is increased and the molecules are stretched [41], increasing the opportunities for hydrophobic interactions with surfactants. Therefore, the cmc values of GTA-m-CS decrease with increasing temperature. The higher the concentration of GTA-m-CS, the more functional groups involved in the hydrophobic interaction, resulting in lower cmc values.

The cmc values of DDEAC obtained by surface tension were different from those obtained by electrical conductivity, which was caused by different working principles of the two methods. The cac values of these systems are absent in the plots of conductivities, which ascribed to the large concentration gradient.

The degree of counterion binding to micelle (β) is one of the important parameters during micellization processes, which could be calculated from the degree of counterion dissociation (α). The values of α could be obtained from the ratio of the slopes above and below cmc, and β=1−α. The value of β is dependent on the size of micelle. A large micelle is prone to attract more counterions, corresponding to a large β value. Table 3 shows that the β value of DDEAC is much lower than those of GTA-m-CS/DDEAC systems, indicating that the GTA-m-CS/DDEAC micelles are larger than that of DDEAC, and the micelle size of GTA-m-CS/DDEAC is almost unaffected by GTA-m-CS concentration. This is ascribed to the hydrophobic interaction between DDEAC and methyl groups in $–{N(\mathrm{CH}}_{3}{)}_{3}^{+}$, and the electrostatic repulsion between the $–{N(\mathrm{CH}}_{3}{)}_{3}^{+}$ groups [24]. The result is consistent with that glycidyl trimethyl ammonium chloride-modified chitosan/1-tetradecyl-3-methylimidazolium bromide system [24].

Thermodynamic parameters of micelle processes, such as standard Gibbs free energy ($\Delta G_{m}^{0}$), standard enthalpy change ($\Delta H_{m}^{0}$) and standard entropy change ($T\Delta S_{m}^{0}$), could be obtained based on the phase separation model

(5) $$\Delta G_{m}^{0}=\left(1+\beta \right)RTlnX_{cmc}$$

(6) $$\Delta H_{m}^{0}=-RT^{2}\left(0.5+\beta \right)\frac{lnX_{cmc}}{dT}$$

(7) $$T\Delta S_{m}^{0}=\Delta H_{m}^{0}-\Delta G_{m}^{0}$$

where $X_{cmc}=\frac{cmc}{55.4}$, 55.4 is the number of moles in 1 L water, the unit of cmc is mol/L.

All the Gibbs free energy ($\Delta G_{m}^{0}$) are negative (Table 3), indicating that the formation of DDEAC micelles is spontaneous. The Gibbs free energy decreases with increasing temperature and GTA-m-CS concentration, suggesting a high temperature and GTA-m-CS concentration are beneficial to the formation of DDEAC micelles. On one hand, this ascribes to a high temperature promoting the movement of DDEAC molecules, which is conducive to its hydrophobic interaction with neighboring molecules. On the other hand, however, GTA-m-CS with a high concentration can provide more hydrophobic groups to interact with DDEAC molecules [42,43].

The values of standard enthalpy change are positive, indicating the micellar formation process is endothermic [25]. The values first increase with rising temperature and then decrease, suggesting the main intermolecular force between GTA-m-CS and DDEAC changes from hydrophobic interaction to electrostatic interaction. The temperature that corresponds to the maximum $\Delta H_{m}^{0}$ decreases with the increase of GTA-m-CS concentration. The result is consistent with that of the degree of counterion binding to micelle. By comparing the values of $\Delta H_{m}^{0}$ and $T\Delta S_{m}^{0}$, it can be concluded that the micelle formation is an entropy-driven process, which is different from the micellar formation process of pure DDEAC [25].

### 3.5. Steady-State Fluorescence

**Figure 5:**
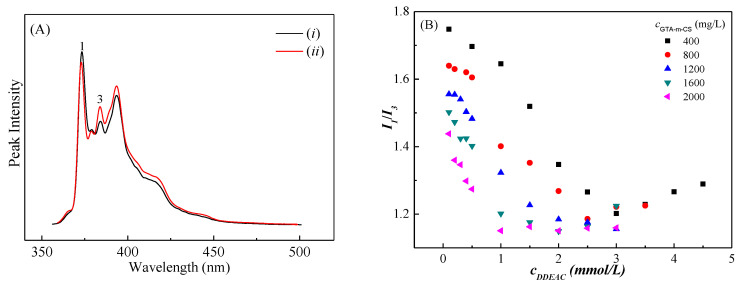
Plots of steady state fluorescence emission spectra (**A**) for GTA-m-CS/DDEAC solutions with DDEAC concentration lower (*i*) and higher than cmc (*ii*), and *I*_1_/*I*_3_ against DDEAC concentration (**B**) at 25 °C.

The steady-state fluorescence of GTA-m-CS/DDEAC system could also reflect the formation of micelles while pyrene is used as a probe. There are five characteristic emission bands in pyrene fluorescence spectrum within 370 to 400 nm (Figure 5A). The intensity of peak 3 is significantly affected by the environment where the pyrene molecules reside. It increases while the environment is transferred from polarity to non-polarity. With the increase of DDEAC concentration, the micelles begin to form, and pyrene molecules were solubilized. The intensity of peak 3 increases (sample *ii* in Figure 5A). From the plot of *I*_1_/*I*_3_ ratio against DDEAC concentration (Figure 5B), the critical micellar concentrations of 2.99 ($c_{\mathrm{GTA}-m-\mathrm{CS}}=400\;\mathrm{mg}/L$), 2.51 (800 mg/L), 1.99 (1200 mg/L), 1.49 (1600 mg/L) and 1.00 (2000 mg/L) mmol/L are obtained. The values are consistent with those obtained from the conductivity method, and are slightly different from those obtained by the surface tension method, which is attributed to the surface activity of GTA-m-CS.

The aggregation number (*N*_agg_) of DDEAC in micelles could be calculated from the relationship between the concentration of benzophenone (quenching agent) and the intensity of peak 3. To maintain the stability of the micelles, the concentrations of DDEAC were 10 times its cmc. The linear equations of ln(*I*_0_/*I*) against quenching agent (*c*_Q_) concentration for different GTA-m-CS/DDEAC system are as follows. The aggregation numbers are calculated as 6.26 ($c_{\mathrm{GTA}-m-\mathrm{CS}}=400\;\mathrm{mg}/L$), 5.91 (800 mg/L), 7.11 (1200 mg/L), 6.68 (1600 mg/L) and 6.75 (2000 mg/L), which are much less than pure DDEAC micelle (ca. 49) [25]. This is ascribed to the amphiphilic properties of GTA-m-CS, hydrophobic and electrostatic interactions between GTA-m-CS and DDEAC.

(8) $$\ln\frac{I_{0}}{I}=\frac{6.26\cdot c_{Q}}{c_{\mathrm{DDEAS}}-\mathrm{cmc}}-0.14\;\left(R^{2}=0.99)\;(c_{\mathrm{GTA}-m-\mathrm{CS}}=400\;\mathrm{mg}/L\right)$$

(9) $$\ln\frac{I_{0}}{I}=\frac{5.91\cdot c_{Q}}{c_{\mathrm{DDEAS}}-\mathrm{cmc}}-0.03\;(R^{2}=0.98)\;(c_{\mathrm{GTA}-m-\mathrm{CS}}=800\;\mathrm{mg}/L)$$

(10) $$\ln\frac{I_{0}}{I}=\frac{7.10\cdot c_{Q}}{c_{\mathrm{DDEAS}}-\mathrm{cmc}}+0.01\;(R^{2}=0.99)\;(c_{\mathrm{GTA}-m-\mathrm{CS}}=1200\;\mathrm{mg}/L)$$

(11) $$\ln\frac{I_{0}}{I}=\frac{6.68\cdot c_{Q}}{c_{\mathrm{DDEAS}}-\mathrm{cmc}}+0.04\;(R^{2}=0.99)\;(c_{\mathrm{GTA}-m-\mathrm{CS}}=1600\;\mathrm{mg}/L)$$

(12) $$\ln\frac{I_{0}}{I}=\frac{6.75\cdot c_{Q}}{c_{\mathrm{DDEAS}}-\mathrm{cmc}}-0.02\;(R^{2}=0.98)\;(c_{\mathrm{GTA}-m-\mathrm{CS}}=2000\;\mathrm{mg}/L)$$

### 3.6. Curcumin Encapsulation and Releasing

**Figure 6:**
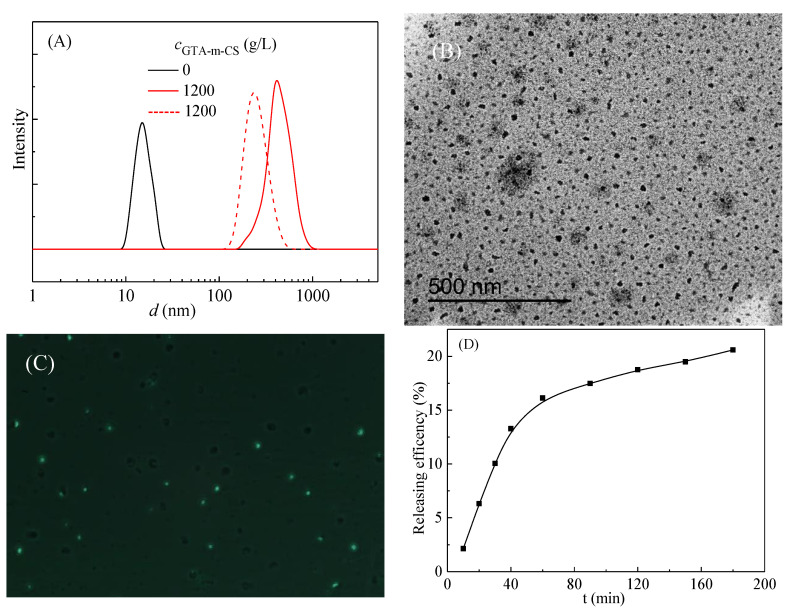
Hydrodynamic radius (**A**), TEM images (**B**), fluorescence confocal micrograph (**C**) and curcumin-releasing behavior (**D**) of curcumin-loaded DDEAC and GTA-m-CS/DDEAC micelles.

At fixed concentrations of DDEAC (3 mmol/L) and GTA-m-CS (1200 g/L), the hydrodynamic radius of curcumin loaded micelles increase from 5 nm [25] and 234 nm (dash line in Figure 6) to 15 nm and 420 nm (solid red line in Figure 6A), indicating the residence of curcumin in micelles. The TEM images of curcumin loaded GTA-m-CS micelles confirm the formation of micelles (Figure 6B), which diameter ranges from 42.86 nm to 83.04 nm with an average diameter of 61.89 nm. The diameter is much smaller than that measured by DLS, which is attributed to the different working principles of the two methods. Fluorescence confocal microscopic (Figure 6C) shows that the curcumin-loaded micelles are monodispersed. The zeta potential of GTA-m-CS/DDEAC complex decreases slightly from 28.32 ± 3.57 mV to 20.13 ± 2.69 mV after the encapsulation of curcumin. This is attributed to the hydrophobic interactions between curcumin and DDEAC and –CH_3_, shielding the positive electricity of $–N{\left({\mathrm{CH}}_{3}\right)}_{3}^{+}$. The curcumin encapsulation efficiency (CEE) is 31.63 ± 2.37%, which is lower than that encapsulated in Tween 85 [44], Pluronic F127 [45] and other nanoemulsion systems [46]. This is due to the competitive interaction between curcumin, DDEAC and GTA-m-CS.

Figure 6D shows that the encapsulated curcumin shows a rapid release rate for the first 60 min, and the releasing efficiency reaches 16.13%. The curcumin is then released slowly until the end of the experiment, and the maximum releasing efficiency is 20.59%. Curcumin is a natural compound with good anti-inflammatory and anti-cancer properties with low toxicity and small adverse reactions. It has a wide range of pharmacological activities, including anti-inflammatory, antioxidant, lipid-regulating, anti-viral, anti-infection, anti-tumor, anticoagulant, anti-liver fibrosis and anti-atherosclerosis. However, curcumin is insoluble in water, which makes it difficult to play a pharmacological role in daily life. The encapsulation and sustained release of curcumin via GTA-m-CS/DDEAC system will expand the application of curcumin in medicine and functional food. Research in these areas will be continued.

## 4. Conclusions

GTA-m-CS and DDEAC were successfully synthesized through the nucleophilic substitution method. The DDEAC began to interact with GTA-m-CS through hydrophobic interaction at its concentration of ca. 0.088 mmol/L at room temperature, which was almost unaffected by GTA-m-CS concentration. Both the cmc and γ_cmc_ values of DDEAC increased with the increase of GTA-m-CS concentration, and decreased with raising temperature. The efficiency of GTA-m-CS/DDEAC in reducing surface tension was much higher ($pc_{20}=0.012\sim 0.078$ mmol L) than that of DDEAC ($pc_{20}=0.20$ mmol L) for the good synergistic effect. The ability to reduce surface tension ($\pi_{\mathrm{cmc}}=41\sim 45$ mN/m) was only slightly affected by the synergistic effect. The formation of GTA-m-CS/DDEAC aggregates was spontaneous and an endothermic process, and the number of DDEAC in one GTA-m-CS/DDEAC aggregate ranged from 6 to 10, depending on the concentration of GTA-m-CS. With the increase of DDEAC concentration, the hydrophobic interaction between DDEAC and GTA-m-CS induces the crimp of GTA-m-CS molecules, and reduces the viscosity of the system. The curcumin encapsulating efficiency of GTA-m-CS/DDEAC aggregates reaches 31.63 ± 2.37%, and could sustain release 20.59% in 180 min. The results mean that GTA-m-CS/DDEAC system will expand the application of curcumin in medicine and functional food.

## Figures and Tables

**Table 1:** Parameters of GTA-m-CS/DDEAC Systems with Different Concentration of GTA-m-CS at 20 °C.

*c*_GTA-m-CS_ (mg/L)	cmc (mmol/L)	γ_cmc_ (mN/m)	*p*c_20_ (mmol/L)	*π*_cmc_ (mN/m)
0	1.13	27.26	0.20	45.02
400	1.32	26.87	0.012	45.41
800	1.41	29.42	0.078	42.86
1200	1.51	27.78	0.056	44.50
1600	1.58	28.77	0.020	43.51
2000	1.64	30.64	0.047	41.64

**Table 2:** Critical Micelle Concentration and Surface Tension of GTA-m-CS/DDEAC Systems with GTA-m-CS Concentration of 1200 mg/L and Different Temperature.

*t* (°C)	cmc (mmol/L)	*γ*_cmc_ (mN/m)
20	1.51	27.78
30	1.39	27.70
40	1.16	26.14
50	0.96	25.61
60	0.83	24.63

**Table 3:** Parameters of GTA-m-CS/DDEAC Systems with Different Concentration of GTA-m-CS and Temperature Obtained from Conductivity Curves.

*c*_GTA-m-CS_ (mg/L)	*T* (°C)	cmc (mmol/L)	*β*	$\Delta G_{m}^{0}\;(\mathrm{kJ}/\mathrm{mol})$	$\Delta H_{m}^{0}\;(\mathrm{kJ}/\mathrm{mol})$	$T\Delta S_{m}^{0}\;(\mathrm{kJ}/\mathrm{mol})$
400	20	2.67	0.62	−40.94	4.63	45.57
	30	2.52	-	−42.59	4.03	46.62
	40	2.43	-	−44.15	5.58	49.73
	50	2.23	-	−45.95	5.95	51.90
	60	2.15	-	−47.54	3.77	51.32
800	20	2.36	0.61	−41.45	3.46	44.92
	30	2.26	-	−43.05	10.41	53.46
	40	1.85	-	−45.35	14.92	60.27
	50	1.63	-	−47.37	7.06	54.43
	60	1.60	-	−48.93	1.92	50.85
1200	20	1.89	0.69	−42.37	12.34	54.71
	30	1.62	-	−44.47	13.45	57.92
	40	1.38	-	−46.64	11.48	58.12
	50	1.26	-	−48.54	8.44	56.98
	60	1.16	-	−50.43	8.55	58.98
1600	20	1.47	0.61	−43.40	4.48	47.88
	30	1.39	-	−45.12	7.98	53.10
	40	1.22	-	−47.18	6.71	53.89
	50	1.20	-	−48.76	3.30	52.06
	60	1.14	-	−50.51	5.30	55.82
2000	20	1.28	0.61	−43.97	15.85	59.82
	30	1.05	-	−46.32	11.87	58.18
	40	0.97	-	−48.19	4.57	52.76
	50	0.95	-	−49.83	2.05	51.88
	60	0.93	-	−51.47	2.20	53.67

Note: *β*-degree of counterion binding to micelle; “-” –not determined.
